# Correlation between Perturbation of Redox Homeostasis and Antibiofilm Capacity of Phytochemicals at Non-Lethal Concentrations

**DOI:** 10.3390/antiox11122451

**Published:** 2022-12-12

**Authors:** Michael S. Christodoulou, Federica Villa, Andrea Pinto, Francesca Cappitelli

**Affiliations:** Dipartimento di Scienze per gli Alimenti, la Nutrizione e l’Ambiente, Università degli Studi di Milano, Via Celoria 2, 20133 Milan, Italy

**Keywords:** biocide-free antibiofilm substances, phenolic phytochemicals, sub-lethal concentrations, reactive oxygen species, antioxidants and pro-oxidants

## Abstract

Biofilms are the multicellular lifestyle of microorganisms and are present on potentially every type of biotic or abiotic surface. Detrimental biofilms are generally targeted with antimicrobial compounds. Phytochemicals at sub-lethal concentrations seem to be an exciting alternative strategy to control biofilms, as they are less likely to impose selective pressure leading to resistance. This overview gathers the literature on individual phytocompounds rather than on extracts of which the use is difficult to reproduce. To the best of our knowledge, this is the first review to target only individual phytochemicals below inhibitory concentrations against biofilm formation. We explored whether there is an overall mechanism that can explain the effects of individual phytochemicals at sub-lethal concentrations. Interestingly, in all experiments reported here in which oxidative stress was investigated, a modest increase in intracellular reactive oxygen species was reported in treated cells compared to untreated specimens. At sub-lethal concentrations, polyphenolic substances likely act as pro-oxidants by disturbing the healthy redox cycle and causing an accumulation of reactive oxygen species.

## 1. Sub-Lethal Concentrations of Antibiofilm Phytochemicals as an Innovative Strategy against Microbial Resistance

Biofilms are the multicellular lifestyle of microorganisms and are present on potentially every type of biotic or abiotic surface. Some biofilms are beneficial to humans in a variety of fields, including energy production [1], bioremediation [2] and organism health [3]. In contrast, other biofilms are a threat to humans [4] and other organisms [5], and to human-made systems [6]. Focusing on the underlying mechanisms of biofilm formation in response to environmental cues is pivotal, as a critical characteristic of detrimental biofilms is their extremely enhanced capability to resist antimicrobial agents in comparison to planktonic cells.

Antimicrobial agents are the traditional approach to combat biofilms that cause deterioration or diseases or that compromise functional performance within an industrial setting. However, biofilms can develop resistance to antimicrobials, meaning that a substance is no longer effective in targeting the detrimental biological agents [7,8]. For instance, some *Listeria monocytogenes* strains are now increasingly resistant due to the excessive use of antimicrobial agents in animal and food production and human medicine [9]. The idea that is emerging is one of simply disarming detrimental microorganisms rather than killing them, to avoid antimicrobial resistance.

**Figure 1:**
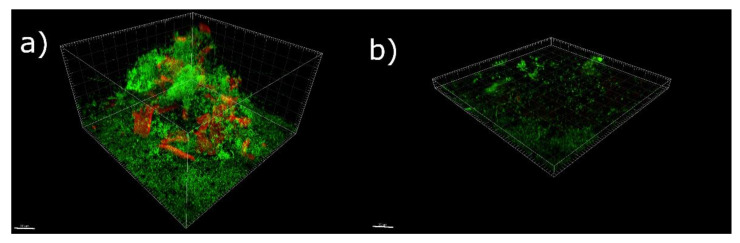
Side views of 3D reconstructed CLSM images of biofilm grown without (**a**) and with (**b**) salicylic acid (λ_ex_ at 488 nm, and λ_em_ < 530 nm, 60×, 1.0 NA water immersion objective). Live cells were stained green with Syber green I, whereas the polysaccharide matrix was stained red with Texas Red-labeled ConA. Scale bar = 30 μm.

Many natural bioactive substances targeting virulence/detrimental factors seem to be an exciting strategy to control biofilms, since they are less likely to impose selective pressure leading to resistance, as they can work as biocide-free agents [10]. This strategy addresses the sustainable management of biofilm challenges. Many organisms produce antibiofilm compounds that work at sub-lethal concentrations, e.g., frog-skin-derived Temporin L on *Pseudomonas fluorescens* [11], marine-derived fungi secalonic acid D and B on *Staphylococcus aureus* biofilm [12], and reuterin from strains of *Lactobacillus reuteri* on *Clostridium perfringens* [13]. However, the selection of phytocompounds able to alter microbial lifestyle is opportune and relevant, since plant species naturally produce an extremely wide range of secondary metabolites used as a defense against environmental microorganisms [14]. Sub-lethal concentrations mean that the minimum inhibitory concentration (MIC, mg/mL) has been ascertained and the antibiofilm efficacy of a phytochemical is measured at lower doses [15] (Figure 1). Careful attention should be paid to rates of application, as some literature compares the effects of compounds at the same quantity, e.g., in Jagani et al. [16], who used 8 µg of phenolic compound per milliliter of solution, which led to some compounds being used at sub-MIC concentrations and others above MIC.

Different compounds have different MICs against specific target microorganisms. In the work by Rózalska and colleagues [17], ursolic acid always showed a lower MIC than ellagic acid, epicatechin and quercetin when *S. aureus* ATCC 43300, *S. aureus* H9, *Candida albicans* ATCC 10231 and *C. albicans* A4 were considered. A concentration below the MIC (4 μg/mL) of ellagic acid reduced biofilm development of *Streptococcus dysgalactiae* NCTC 4671 up to 27% and *S. dysgalactiae* ATCC 27957 up to 21%, but 4 μg/mL had no effect on *S. aureus* biofilm growth [18]. In addition, even with the same phytocompound, it is not always possible to gather unique conclusions as different investigations were often carried out with variations in the methodologies, i.e., differences in sub-lethal doses, microbial targets, microbial cell density, and culture media [19]. Time could also be a key factor. Some plant-derived compounds were proven to exhibit a time-dependent antibiofilm effect. For instance, cannabidiol at the sub-MIC dose of 6.25 μg/mL did not decrease the biofilm mass after 1 day, while inhibition of biofilm mass by 28% and 39% was seen after 2 and 3 days, respectively [20]. Antibiotics alone at sub-MIC doses are claimed to promote biofilm formation and the development of antibiotic resistance [21]. The synergic effect of phytochemicals at sub-lethal doses and drugs has been tested and found very promising. Thyme oil and its major component thymol displayed significant synergy with fluconazole against *Candida* biofilms. The findings were remarkably relevant, as the above drugs, when used alone, may require too high doses, resulting in increased adverse side effects such as in vivo toxicity [22]. Similarly, non-lethal zosteric acid concentrations strongly enhanced sensitivity towards chlorhexidine digluconate, with most of the *Candida* cells being killed within 2 h [23]. Upon 40 μg/mL quercetin treatment, *Klebsiella pneumoniae*, *P. aeruginosa* and *Yersinia enterocolitica* displayed a >90% increase in sensitivity to tetracycline, kanamycin, and gentamycin [24]. According to Abdelraheem et al. [19], vitamin C (ascorbic acid) had a synergistic effect with antibiotics against *P. aeruginosa* both in vitro and in vivo. The authors suggested that vitamin C should be consistently prescribed with antibiotics to treat *Pseudomonas* infections to reduce treatment duration and antibiotic dose.

Some papers report the employment of plant extracts. The violacein production in *Chromobacterium violaceum* 12472 was reduced by more than 80% by using an extract of *Plumbago zeylanica*, without inhibiting planktonic cell growth [25]. In the above research, as in others involving extracts [26,27], many plant metabolites were detected [28,29,30] but the isolation and study of the most bioactive compounds were not always pursued. Essential oils are also used at sub-lethal concentrations, but they are not always composed of a single compound [31].

Many studies on the effects on biofilms of phytochemical treatment at sub-MICs were retrieved from the literature, and a few are listed in Table 1. Importantly, the characteristics of extracts are largely influenced by the source and the extraction method used [32] and it is often impossible to reproduce the same results in other laboratories. Consequently, in Table 1 as well as in the list of individual phytochemicals below, only the literature considering the investigation of individual plant compounds is reported.

For some individual phytocompounds, specific effects have been reported that did not compromise the microbial growth of the target organism while exhibiting antibiofilm activity. The following alterations were detected after exposure to the plant-based compound below the MIC: (1) QS (cinnamaldehyde in Niu et al. [56]; tannic acid, ellagic acid and (−)-epigallocatechin gallate in Huber et al. [57]; cyanidin in Gopu and Shetty [37]; caffeine in Maisarah Norizan et al. [58]); (2) ability to adhere to a surface (shikonin in Li et al. [15]); (3) mobility (piperine in Das et al. [47]); (4) production of extracellular polymers (limonene and beta-caryophyllene in Tomaś et al. [59]); (5) translocation apparatus (glycone (myricitrin, hesperidin and phloridzin) and aglycone flavonoids (myricetin, hesperetin and phloretin) in Lopes et al. [48]); (6) expression of protective proteins (zosteric acid in Villa et al. [60]) and genes related to pathogenicity (salicylic acid, p-hydroxybenzoic acid, cinnamyl alcohol, p-coumaric acid, and hydrocinnamic acid in Hu et al. [61]); (7) replicational/transcriptional activity (berberine in Wang et al. [41]). Da et al. [62] studied the effects of salicylic acid on *P. aeruginosa* PAO1 and its QS mutant. Interestingly, they reported that the mutant also exhibited a salicylic acid-dependent biofilm inhibition. The researchers suggested that the antibiofilm effect of salicylic acid depended on more than QS disruption and a possible explanation was the iron chelating ability of the phytochemical.

In this review, we want to explore whether there is an overall mechanism that can explain the effects of individual phytochemicals at sub-lethal concentrations.

## 2. Reactive Oxygen Species (ROS)

The atmospheric oxygenation of Earth started ∼2.43 billion years ago [63]. Oxygenic photosynthesis led to the oxidation of reductants. At that time, soluble and relatively bioavailable ferrous iron (Fe^2+^) was the prevailing marine inorganic reductant. Therefore, oceans experienced a 4-fold decrease in iron level due to the insolubility of the oxidized ferric form [64]. Since iron in the ferric form is extremely insoluble, it became scarce and a limiting factor within several ecosystems [65]. Despite the reliance on iron-based chemistry being threatened by oxygenic photosynthesis, contemporary enzymes still rely on iron as a cofactor. Iron is involved in the catalysis of all the redox transitions necessary for sulfur and nitrogen assimilation and many metabolic pathways, e.g., all types of respiration [64].

The increase in O_2_ not only led to changes in metal iron availability but also forced cells to cope with its toxicity. Oxygen has a tendency to acquire electrons from other molecules, although the potency of oxygen to acquire one electron at a time leads, firstly, to the formation of the superoxide anion O_2_^•−^ [65]. The monovalent reduction of oxygen provides the superoxide anion, which is considered both a radical and an anion with the radical sign (•) and a charge of −1 (Equation (1)) [66].

(1) $$O_{2}+e^{-}⇆O_{2}^{•-}$$

Biologically, in respiring organisms, O_2_^•−^ can be generated enzymatically in catalyzed reactions such as in the mitochondrial respiratory chain and in phagocytic nicotinamide adenine dinucleotide phosphate oxidase (NADPH oxidase) [67].

In the second single reduction, the superoxide anion dismutase spontaneously or through enzyme-catalyzed reactions—for example, the superoxide dismutase enzymes (SODs) act to produce hydrogen peroxide (H_2_O_2_) and O_2_ (Equation (2)) [68].

(2) $$2O_{2}^{•-}+2H^{+}\to H_{2}O_{2}+O_{2}$$

Even if the superoxide anion is not considered a strongly oxidizing agent, it is able to univalently oxidize the solvent-exposed [4Fe-4S] cluster of the enzyme dihydroxy acid dehydratase during substrate dehydration. This causes cluster instability, degradation, and loss of the catalytic iron atom, leading to the inactivation of the enzyme [69]. In continuation, the formatted ferrous atom (Equation (3)) is able to reduce the hydrogen peroxide (Equation (2)), based on the Fenton reaction, to form the highly reactive and toxic hydroxyl radical (OH^•^) (Equation (4)). Initially, the Haber–Weiss reaction was considered to be a source of hydroxyl radicals but later this hypothesis was disproved [70]. Although the superoxide anion can reduce aqueous Fe^3+^, even if ferric ions are characterized by poor solubility, to provide Fe^2+^ (Equation (5)), which can enter the Fenton reaction (Equation (4)), the rate of the reduction of Fe^3+^ by the superoxide anion is very slow and for this reason, other cellular reductants are considered for this reaction [71].

(3) $${\left[4\mathrm{Fe}-4S\right]}^{2+}+O_{2}^{•-}+2H^{+}\to {\left[3\mathrm{Fe}-4S\right]}^{+}+H_{2}O_{2}+{\mathrm{Fe}}^{2+}$$

(4) $${\mathrm{Fe}}^{2+}+H_{2}O_{2}\to {\mathrm{Fe}}^{3+}+HO^{•}+HO^{-}$$

(5) $${\mathrm{Fe}}^{2+}+O^{•-}\to {\mathrm{Fe}}^{2+}+O_{2}$$

## 3. Individual Phytocompounds Used at Sub-Lethal Doses

**Figure 2:**
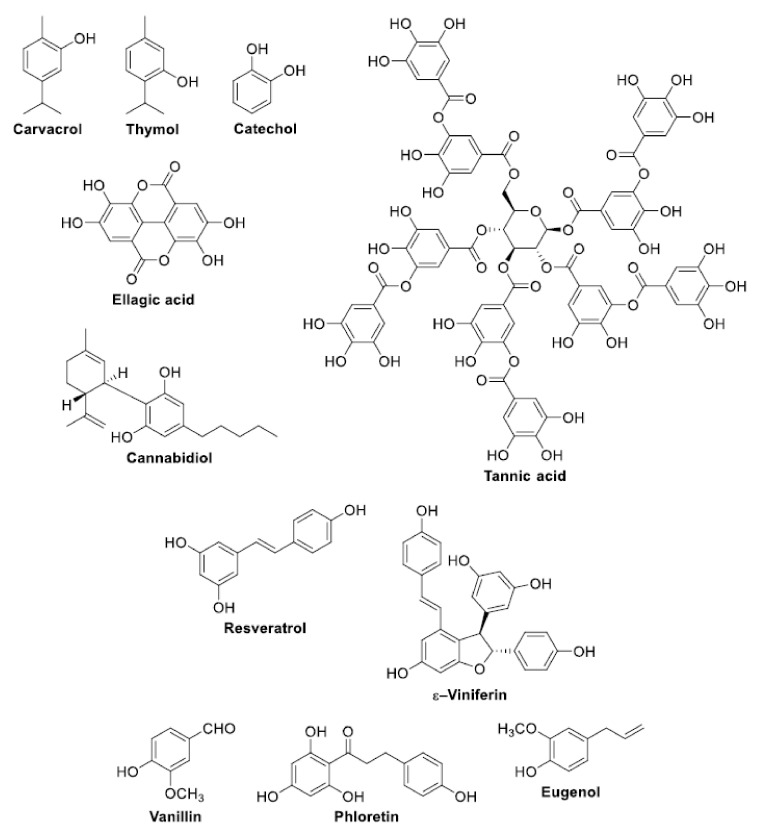
Structures of phenols.

**Figure 3:**
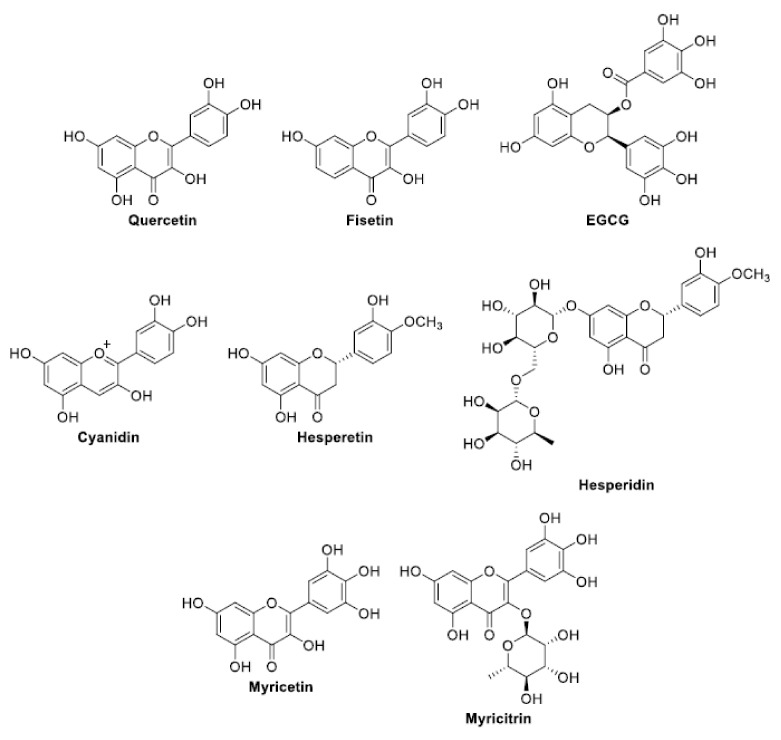
Structures of flavonoids.

**Figure 4:**
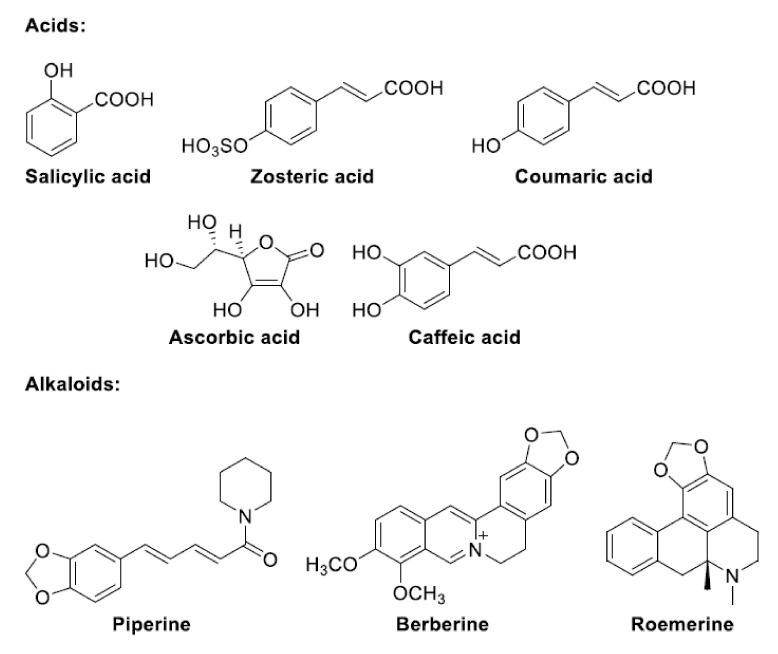
Structures of acids and alkaloids.

**Figure 5:**
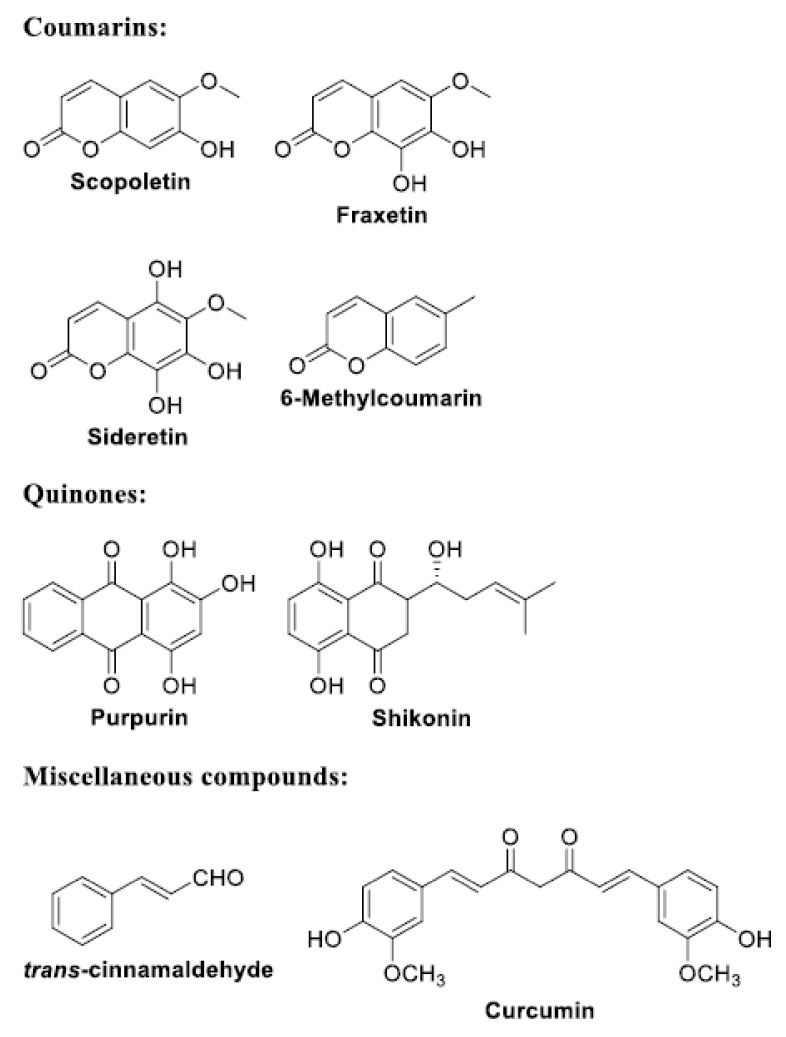
Structures of coumarins, quinones and miscellaneous compounds.

The following is a list of antibiofilm compounds used at sub-lethal doses. The chemical structure of these compounds is reported in Figure 2, Figure 3, Figure 4 and Figure 5. The antioxidant activity of compounds is generally evaluated in multiple radical scavenging assay systems such as 2,2′-diphenyl-1-picryl-hydrazyl (DPPH•), 2,2′-azinobis(3-ethylbenzothiazoline-6-sulfonic acid (ABTS^•+^), hydroxyl radical, superoxide anion, hydrogen peroxide, the Oxygen Radical Absorbance Capacity (ORAC), lipid peroxidation, galvinoxyl, and linoleic acid peroxidation. The chelating ability of the compounds for Fe^2+^ is measured by the ferrozine assay.

### 3.1. Phenols (Figure 2)

#### 3.1.1. Carvacrol and Thymol

Considering that microbial response to environmental stimuli can be dose dependent is extremely important. While repressing biofilm formation, sub-inhibitory concentrations of carvacrol and thymol (in the essential oil of oregano) led to treated *E. coli* O157:H7 cells upregulating the genes related to membrane, heat, and oxidative stress responses and iron uptake, thus increasing direct and cross-resistance [72]. At 0.33 mM sub-lethal concentration, both carvacrol and thymol presented antioxidant activity in the linoleic acid emulsion assay, with values of 95.3% and 96.8%, respectively, but almost no inhibition in the DPPH• assay at the same concentration [73].

#### 3.1.2. Cathecol

Cathecol is strictly linked to salicylic acid (SA), as the enzyme salicylate monooxygenase converts salicylate into catechol. The PGPR *Bacillus subtilis* protects plant roots from pathogenic bacteria, also forming biofilms. The non-colonization and suppression of *B. subtilis* biofilm formation on the roots of *A. thaliana* line *NahG*, a transgenic line-containing gene for salicylate hydroxylase, which hydrolyzes SA and results in the overproduction of catechol, were observed [74]. The authors speculated that *B. subtilis* colonization and biofilm inhibition was due to the high ROS concentrations generated by catechol augmentation on *NahG* roots. The antioxidant activity of catechol is associated with the consecutive two-electron oxidation of the compound to form the corresponding quinone [75]. Moreover, catechol is capable of forming complexes with Fe(III), and its stoichiometry depends on the pH at which the reaction is performed. However, if the complexes undergo an internal redox reaction giving rise to iron (II) and quinone, iron (II) will produce hydroxyl radicals through the Fenton reaction [76].

#### 3.1.3. Tannic Acid and Ellagic Acid

A concentration of 20 μg/mL (11.8 µM) of tannic acid was found to strongly inhibit the biofilm formation of *S. aureus* [77]. Auto-oxidation of catechins leads to their polymerization and forms tannins [51]. Tannic acid presented good radical scavenging potential, very near to sub-lethal concentrations, with 50% of radical scavenging to be achieved on 20.42 and 20.19 µM in the DPPH• and superoxide assay, respectively. Moreover, tannic acid showed 50% ferrous chelation at 11.63 µM, with the highest dose of tannic acid inhibiting complex formation up to 77% [78]. The plant phenolics ellagitannins encompass more than 1000 identified natural bioactive compounds [79]. In the study by Dalvi and colleagues [80], spectral analyses of iron-ellagic acid complexes showed that ellagic acid seizes ferric ions from EDTA within hours, and from citrate within 1 min. In fact, ellagic acid showed a moderate radical scavenging potential but was found to induce a dose-dependent inhibition of the ferrozine-Fe^2+^ complex formation up to 70% at the highest dose of 100 μM [81].

#### 3.1.4. Cannabidiol

At sub-lethal doses, cannabidiol showed a prevention and control activity against *C. albicans* SC5313 biofilm [20]. Cannabidiol modified mitochondrial activity and induced intracellular ROS production [20]. Cannabidiol was able to reverse iron-induced expression of the mitochondrial fission protein DNM1L in rats [82].

#### 3.1.5. Resveratrol and ε-Viniferin

Resveratrol occurs in grapes and grape-derived beverages such as red wine. A statistically significant and concentration-dependent effect was proved for resveratrol with >80% reduction for all *Propionibacterium acnes* strains studied without affecting planktonic cell growth [53]. The mechanism by which resveratrol exerts its activity was not investigated. Sub-MICs of *trans*-resveratrol and ε-viniferin, a resveratrol dimer, inhibited the biofilm formation of *P. aeruginosa* PAO1 and PA14 [83]. In particular, *trans*-resveratrol at 50 μg/mL (219 µM) decreased *P. aeruginosa* PAO1 biofilm formation by 92%, and ε-viniferin at 50 μg/mL (110 µM) decreased *P. aeruginosa* PA14 biofilm formation by 82%. The ability of *trans*-resveratrol and ε-viniferin to scavenge the hydroxyl radical formation was determined in the 2-deoxyribose degradation and in the rat liver microsomal lipid peroxidation assays. At sub-lethal concentrations, *trans*-resveratrol, *trans*-ε-viniferin and *cis*-ε-viniferin were able to inhibit 50% of the produced radicals at 7.35, 0.17 and 0.43 µM, respectively, in the 2-deoxyribose degradation assay and 2.58, 0.41 and 1.08 µM, respectively, in the lipid peroxidation assay [84]. Additionally, 50% of scavenging activity on the superoxide radicals of *trans*-ε-viniferin was obtained at 140 µM [85]. *Trans*-resveratrol, which is by far the most potent chelator of copper, does not chelate iron [86].

#### 3.1.6. Eugenol

In addition to inhibiting biofilm formation, eugenol at sub-MIC concentrations inhibited the production of virulence factors, including pyocyanin and pyoverdine, in *P. aeruginosa* PAO1 [55,87]. Eugenol inhibited the generation of the superoxide anion by 50% at a sub-lethal concentration of 250 µM and the generation of hydroxyl radicals to an extent of 70% at 200 µM [88]. In the DPPH• assay, 50% of the radical scavenging activity of eugenol was obtained at 510 µM [89].

#### 3.1.7. Vanillin

Reduced production of ergosterol in presence of sub-MIC vanillin indicated that inhibition of ergosterol biosynthesis may be a probable target in *C. albicans* [34] and modulation of ergosterol content had been already proven critical in adaptation to oxidative stress [90]. Vanillin showed no activity in the DPPH• radical, ORAC and galvinoxyl radical scavenging assays but showed stronger activity than ascorbic acid and Trolox in the ABTS^•+^ scavenging assay with a sub-lethal concentration at 19.4 μM for the effective scavenging of 50% of the formatting radicals [91]. On the other hand, vanillin did not display any Fe^2+^ chelating ability [92].

#### 3.1.8. Phloretin

For 50% of the radicals formed, the scavenging ability of phloretin was found at 63.5, 108.4 and 4.3 µM concentrations in the DPPH•, superoxide anion and ABTS^•+^ assays, respectively [93]. In addition, phloretin showed 50% ferrous chelation at 162 µM, with the highest metal chelating activity of 89.23% at 292 µM [94].

### 3.2. Flavonoids (Figure 3)

#### 3.2.1. Quercetin and Fisetin

In the study carried out by Lee et al. [77], only 1 μg/mL of quercetin inhibited biofilm formation by methicillin-resistant *S. aureus* by >80% and methicillin-sensitive *S. aureus* strains ATCC 6538 and ATCC 25923 by >50%. In a later investigation, upon treatment with sub-MIC quercetin, overexpression was proved for the proteins DnaK, EF0080 and OsmC, showing that *E. faecalis* cells were under oxidative and general stress [39]. In addition, the NADH peroxidase (*npr*), an oxidoreductase enzyme playing a critical role in maintaining cellular redox homeostasis, was overexpressed as well as chaperone protein DnaK and chaperonin GroS, both proteins involved in stress management [39]. Quercetin can completely suppress Fenton chemistry both at sub-lethal micromolar levels (10 µM concentration) and in the presence of major cellular iron chelators such as ATP or citrate in hydroxyl radical formation based on the 2-deoxyribose degradation assay. However, the radical scavenging activity of quercetin provides only partial protection against Fenton chemistry-mediated damage while Fe chelation by quercetin can completely inhibit Fenton chemistry, indicating that the chelation may be key to its antioxidant activity [95]. Fisetin (5-deoxyquercetin) is a flavonoid commonly found in several fruits and legumes. At a very low concentration (16 μg/mL, 55.9 µM), fisetin inhibited the biofilm development of *S. aureus* 8325 by 90% [18]. Fisetin increased its radical scavenging percentages in a dose-dependent manner in various antioxidant assays at sub-lethal concentrations. The values for the 50% radical inhibition in hydroxyl, superoxide, DPPH• and ABTS^•+^ scavenging assays were 47.41 ± 4.50, 34.05 ± 0.87, 9.69 ± 0.53 and 2.43 ± 0.14 μM, respectively [96].

#### 3.2.2. (−)-Epigallocatechin-3-gallate

(−)-Epigallocatechin-3-gallate (EGCG), which is the major polyphenolic component of tea, decreased *Vibrio mimicus* autoaggregation and swimming motility, favored membrane permeability and ROS production led to cell membrane damage and caused potassium leakage [49]. Approximately at tea cup amount (200 μg/mL, 436 µM), EGCG eliminates *Escherichia coli* K12 biofilm matrix by interfering with the assembly of curli subunits into amyloid fibers and by provoking the σ^E^ cell envelope stress response [50]. Interestingly, this phytocompound can sometimes promote biofilm formation and tolerance to specific antibiotics [97]. The concentration of EGCG for 50% radical scavenging was decreased with decreasing pH value in the DPPH• assay. At pH 10 the scavenging activity was 0.7 µM, at pH 7 of 0.6 µM, and at pH 4 of 1.4 µM, albeit in sub-lethal doses [98]. Additionally, EGCG presented a 50% radical scavenging at concentrations of 1.8 and 1.0 µM against the superoxide anion and hydroxyl radical, respectively [99]. Moreover, EGCG might have iron-chelating activity since it was able to inhibit paraquat Pq-induced MDA production, at 40 µM concentration, an inhibition that disappeared when excess amounts of FeSO_4_ were added to the reaction mixture [100].

#### 3.2.3. Cyanidin

The anthocyanin cyanidin was proved to affect *K. pneumoniae* strain PUFST23 biofilm development at 50–150 μg/mL (0.17–0.52 mM) [37]. Quercetin and other polyphenols form co-pigments via intermolecular interaction with anthocyanins [101]. Cyanidin and its 3-glucoside are able to chelate iron and reduce both iron and copper [102]. Qian and colleagues [101] evaluated the antioxidant effects of anthocyanin complexes of cyanidin-3-diglucoside-5-glucoside (CY3D5G), rutin and Mg(II)/Fe(III). The antioxidant properties were affected by the molecular combination with CY3D5G-rutin-Fe(III) showing much lower activities than CY3D5G-rutin-Mg(II). Cyanidin and its 3-glucoside showed a dose-dependent antioxidant activity in the DPPH• assay with the 3-glucoside being more potent at sub-lethal concentrations. At 30 µM, the cyanidin-3-glucoside demonstrated the same activity as 30 µM of the positive control Trolox. Additionally, the two compounds exhibited the same dose-dependent activity in the inhibition of the superoxide anion formation with, again, the 3-glucoside being more potent. The cyanidin-3-glucoside at 1 µM concentration displayed a similar inhibition as the superoxide dismutase at 80 mU/mL [103]. Cyanidin presented 50% chelating activity on Fe^2+^ at 60 µM concentration and 60% hydroxyl radical scavenging at 25 µM concentration [104].

#### 3.2.4. Hesperetin, Hesperidin, Myricetin and Myricitrin

Hesperetin and hesperidin showed a significant superoxide radical scavenging effect in a dose-dependent manner at sub-lethal concentrations (40–100 µM), although, at the same range of concentrations, in the DPPH• assay, hesperidin had a slight observed free radical scavenging effect and hesperetin a moderate one. Hesperetin exhibited a weak Fe^2+^ chelating activity at the same concentrations [105]. Myricetin showed a slight radical scavenging effect of 6.2% in the ABTS^•+^ assay at 98 µM [106] but was able to strongly chelate Fe^2+^ at 25 µM [107]. On the other hand, myricitrin exhibited a strong scavenging activity in the DPPH• and hydrogen peroxide assays with 50% of radical inhibition at 3.0 and 65.2 µM, respectively [108].

### 3.3. Acids and Alkaloids (Figure 4)

#### 3.3.1. Salicylic Acid

Mishra and Baek [109] wrote a comprehensive review on salicylic acid (SA). SA is produced by bacteria, fungi and plants. In plants, it is a hormone-regulating plant growth, environmental stress and defense responses against pathogens. At low iron availability in SA-producing bacteria, SA is critical for the biosynthesis of salicyl-derived siderophores or catecholates, e.g., anachelin, pyochelin, bacillibactin, petrobactin, enterobactin, photobactin, amychelin, salmochelin, vibriobactin, vulnibactin and mycobactin. The antioxidant activity of SA is based on the redox deactivation of iron through chelation and not by hydroxyl radical scavenging. The voltametric results indicated that the iron-salicylate complex does not have the thermodynamic driving force to act as an effective Fenton reagent necessary for the production of damaging oxygen-containing radicals [110]. In the DPPH• assay, salicylic acid did not exhibit any antiradical activity even at concentrations of 800 µmol/assay [111].

#### 3.3.2. Zosteric Acid

After proving its ability to contrast biofilm formation at sub-lethal doses [23], zosteric acid or p-(sulfoxy)cinnamic acid, made by the seagrass *Zostera marina*, was shown to interact with the *E. coli* protein NADH:quinone reductase, WrbA [112]. Investigation of the antibiofilm activity revealed that the para-sulfoxy ester group is not responsible, whereas the cinnamic acid scaffold carries the antibiofilm performance. A study by Kurth et al. [113] demonstrated that the biofilm-inhibiting effects of zosteric acid on *Vibrio natriegens* can be entirely attributed to coumaric acid, which is released from zosteric acid by sulfatase activity. The scavenging ability of coumaric acid in the DPPH•, hydrogen peroxide, superoxide and ABTS^•+^ radical assay, as well as the ferrous chelating ability [114] at concentrations similar to the antibiofilm sub-lethal concentrations of zosteric acid, suggest that zosteric acid could also have an efficient antioxidant activity at sub-MIC.

#### 3.3.3. Ascorbic Acid

The antibiofilm effect of vitamin C on carbapenem-resistant hypervirulent *K. pneumoniae* at sub-MIC was associated with the induction of ROS production, by Xu et al. [38]. Ascorbic acid functions primarily as a donor of single hydrogen atoms, and the radical anion monodehydroascorbate react with radicals. Its crucial role as a reducing agent involves enzymatic reactions, such as for dopamine β-monooxygenase and peptidyl-glycine α-amidating monooxygenase. Additionally, it reduces Fe^3+^ to Fe^2+^ in enzymes that contain Fe^2+^ as a cofactor such as procollagen-proline dioxygenase and procollagen-lysine dioxygenase. Moreover, ascorbate oxidase and ascorbate peroxidase form H_2_O by reducing O_2_ and H_2_O_2_, respectively, by using ascorbate as a single-equivalent donor. The antioxidant and free-radical scavenging activity of ascorbate are associated with its non-enzymatic reduction of superoxide, hydroxyl, alkoxyl, peroxyl, and other radicals [115]. Ascorbic acid is a known potent antioxidant and is used as a positive control in the DPPH• radical scavenging assay and in the ABTS^•+^ radical scavenging assay since the percentage of the antioxidant activity of ascorbic acid at the sub-lethal concentration of 100 µM in both assays is near 100% [116]. In the lipid peroxidation assay, the value of the potency of ascorbic acid to inhibit 50% of the produced radicals was 14 µM [117]. In contrast to its antioxidant use, ascorbic acid under certain conditions can also act as a pro-oxidant and a source of free radicals. The alpha oxo-hydroxy and di-hydroxy ligands in the chemical structure of ascorbic acid create complexes with ferric ions but generally, ascorbic acid is considered a weak chelating agent and cannot form strong iron complexes. Thus, the reduction of ferric ions from ascorbic acid to ferrous irons and ascorbic radical will turn on the cyclic oxidation process [118].

#### 3.3.4. Caffeic Acid

Cattò and colleagues [112] showed that 18.3 µM of caffeic acid reduced *E. coli* adhesion to a hydrophobic surface without killing the cells. The antibiofilm performance of caffeic acid was expressed at a concentration 10-fold lower than that of zosteric acid. The free radical scavenging activity of caffeic acid was near 60% at the sub-lethal concentration in the DPPH• assay [119].

#### 3.3.5. Piperine

Piperine, a bioactive component of pepper, functions as a potent antibiofilm agent to inhibit *S. aureus* biofilm formation by accumulating ROS, but showed no toxicity against WI 38 line at the tested concentrations [47]. Piperine derivatives were investigated as green corrosion inhibitors on iron surfaces, leading to Fe-inhibitor complex in the hetero-atom centers [120].

#### 3.3.6. Berberine and Roemerine

The maximum scavenging concentration of berberine was found at a sub-lethal concentration of 0.76 mM whilst the minimum scavenging activity was found at 5.9 µM in the DPPH• assay, with 50% of radical scavenging at 0.12 mM. In the ABTS^•+^ assay, the free radicals were scavenged in a concentration-dependent manner with the maximum scavenging activity at 1.5 mM and the minimum at 5.9 µM with 50% of radical inhibition at 0.11 mM [116]. The superoxide assay showed 76.3% radical inhibition at 1.5 mM. Roemerine was able to inhibit only 1.8% of the radical generation at 100 µM concentration in the ABTS^•+^ assay, while was not active in the DPPH• assay at the same concentration [116].

### 3.4. Coumarins, Quinones and Miscellaneous Compounds (Figure 5)

#### 3.4.1. Coumarins

More than 1300 coumarins have been identified in plants [36]. Non-grass plants and grass plants possess different iron responses, named Strategy I and Strategy II, respectively, activated in roots under Fe deficiency [121]. The chelation-based mechanism for Strategy II involves the exudation of phytosiderophores that bind ferric Fe in the soil [122]. Then, the complex is taken up by transporters in the root epidermis [121]. The strategy I is a reduction-based mechanism and involves acidification of the rhizosphere via proton secretion, and enzymatic reduction of iron chelates at the root surface [122]. In the reduction strategy, large amounts of coumarins and protons are released in the rhizosphere to reduce Fe^3+^ to Fe^2+^ before transport across the plasmalemma of root epidermal cells [123]. Thus, coumarins (scopoletin, fraxetin and sideretin), improve plant health by engaging microorganisms involved in iron nutrition [124]. Conversely, these specialized metabolites contrast pathogens. 6-methylcoumarin showed significant antibiofilm activity against *P. aeruginosa* PAO1 biofilm at 125 μg/mL [46]. Several virulence factors were inhibited including pyocyanin (which promotes glutathione oxidation that results in ROS formation) and pyoveridine and pyochelin siderophores. Finally, oxidized coumarins are often more reactive than their counterparts, as oxidative hydroxylation and dimerization increase phenolic groups and bring new chelating features [125]. At the sub-lethal concentration of 100 µM, scopoletin and fraxetin were able to inhibit the superoxide anion generation at 100% and 67%, respectively. Moreover, at the same concentration, scopoletin showed a small but significant reduction in the scavenging of hydroxyl radicals [126].

#### 3.4.2. Purpurin

In *C. albicans*, 3 mg/mL (11.7 µM) of purpurin completely inhibited filamentation under most hypha-inducing conditions in most media, downregulating the expression of the hypha-specific genes (qRT-PCR assessment) [52]. The radical scavenging of purpurin was 36.8% in the DPPH•, 83.2% in the linoleic acid peroxidation, 86.8% in the hydrogen peroxide, and 21.1% in the ABTS^•+^ assay at the sub-lethal concentration of 10 µM [127].

#### 3.4.3. Shikonin

Sub-lethal concentrations of shikonin, a naphthoquinone retrieved from the roots of *Lithospermum erythrorhizon*, effectively decreased *L. monocytogenes* biofilm biomass on polystyrene and adherence to glass slides. Further, the transcription of biofilm-associated genes and virulence-associated genes was downregulated/repressed [15]. At sub-lethal concentrations, shikonin was able to inhibit 50% of the radical formation in the superoxide anion, hydroxyl, and *tert*-butylperoxyl radical assays, with values of 7.2, 40 and 27 µM, respectively [128].

#### 3.4.4. *trans*-Cinnamaldehyde

Despite the ability of *trans*-cinnamaldehyde (a major component of bark extract of cinnamon) to suppress biofilm formation, sub-inhibitory concentrations led to *E. coli* O157:H7 treated cells to upregulate the genes related to membrane, heat, and oxidative stress responses and iron uptake hence increasing direct and cross-resistance [72].

#### 3.4.5. Curcumin

The QS-regulated siderophore production in *Aeromonas sobria* was inhibited by 420 μg/mL (1.14 mM) curcumin liposomes (formulated to increase bioavailability of the poor water solubility of curcumin) but not by 280 μg/mL (0.76 mM) free curcumin [54]. Curcumin exposure affected iron homeostasis and oxidative stress response of *P. aeruginosa* PAO1 [129]. In the presence of curcumin, L-ornithine N (5)-monooxygenase, pyoverdine biosynthesis protein and Fe^3+^ pyochelin receptor protein were upregulated, whereas ferroxidase, bacterioferritin, and isochorismate pyruvate lyase were downregulated. These results suggested the insufficient iron acquisition by *P. aeruginosa* PAO1 upon treatment with curcumin. Moreover, curcumin downregulated the expression of the antioxidant enzymes catalase, peroxidase, superoxide dismutase (SOD) and alkyl hydroperoxide reductase, increasing the ROS level. The 50% of the scavenging effect of curcumin on the DPPH• radical assay was achieved at the sub-lethal concentration of 94.6 µM, and curcumin displayed 50% effective radical cation scavenging activity in the ABTS^•+^ assay at 49 µM. The inhibition of superoxide radical generation was 42.7% at 40.7 µM and the scavenging of hydrogen peroxide was 28.4% at the same concentration. Moreover, curcumin exhibited 56.7% ferrous ion chelation at 40.7 µM concentration [130].

## 4. Conclusions

Antimicrobial resistance impacts the achievements of several of the 17 United Nations Sustainable Development Goals (SDGs), in particular SDG 3 ‘Good health and well-being’ (https://sdgs.un.org/goals (accessed on 9 December 2022)). To address the antimicrobial resistance problem, the effects of natural compounds on biofilm formation at sub-lethal concentrations have been extensively investigated. This overview is significant as it gathers together the literature on individual compounds, rather than on extracts, of which the use is difficult to reproduce. Thus, to the best of our knowledge, this is the first review that targets only individual phytochemicals below inhibition concentrations against biofilm formation.

In vitro experiments cited here reported that the phytochemicals are efficient ROS scavengers at sub-MIC antibiofilm concentrations. Due to their features and quantity (production and disposal) inside cells, ROS are recognized as universal signaling chemical species [131]. ROS, either associated with abiotic stresses or produced by host plants, determine the outcome of the plant-bacteria interaction [132], e.g., the optimal formation of legume-rhizobial interaction, especially in the development of symbiosis [131]. It is speculated that a high level of polyphenol oxidase activity in N_2_-fixing nodules relates to the similarity of the response of plants to pathogens since the protective responses of legume plants to the invasion of rhizobia are analogous to the pathogenic process [133]. Ong et al. [134] stated that compounds that could target oxidative stress regulators, such as antioxidants, could potentially be exploited as a novel strategy for biofilm control. General mechanisms of how ROS inhibition influences biofilm growth are still unknown, but mechanisms of how sessile cells cope with the increase in oxidative stress have been reported. For instance, Chua et al. [135] demonstrated that sub-lethal hydrogen peroxide doses are essential for the selection of pro-biofilm-forming pathogenic variants by modulating cyclic-di-GMP levels in *P. aeruginosa*.

Interestingly, in all experiments reported here in which oxidative stress was investigated, a modest increase in intracellular reactive oxygen species was reported in treated cells in comparison with untreated specimens [20,38,47,49]. The above-mentioned increment of ROS is in line with other outcomes obtained with polyphenols [136,137] and could be related to bacterial wall damage. At lethal concentrations, polyphenols cause bacterial wall damage and a subsequent increase in free radicals; this pro-oxidant effect is accompanied by lipid peroxidation and DNA lesions [136]. In order to respond to this damage, the cell starts a self-protective mechanism that could be further enhanced by the antioxidant and radical scavenger activities of the polyphenols themselves [136]. In this respect, one of the major misinterpretations in the field of oxidative stress concerns the scavenging of superoxide (O_2_^•−^) or H_2_O_2_ by small molecules. In fact, the antioxidant enzymes react thousands to millions of times faster with those oxidants than the small molecules, and for this reason, they are the predominant cellular antioxidant mechanism [138]. Polyphenolic antioxidants might act as pro-oxidants by disturbing the healthy redox cycle and causing an accumulation of reactive oxygen species (ROS) (i.e., hydrogen peroxide, superoxide and hydroxyl radicals) [134], whereas a healthy redox cycle promotes microbial attachment, thus favoring biofilm formation [139]. High levels of ROS derived from the presence of polyphenols acting as pro-oxidant compounds were seen to cause the following effects: ascorbic acid was able to suppress the biofilm exopolysaccharide and to inhibit the efflux pump in the KP1088 and HvKP3 *K. pneumoniae* strain by, at the same time, increasing the accumulation of ROS [38]; piperine raised the level of ROS and decreased the bacterial motility of the *S. aureus* (MTCC 96)[47]; an inhibition in the bacterial motility was observed by epigallocatechin gallate in *Vibrio mimicus* accompanied by increased levels of ROS [49]; an accumulation of ROS was detected in the *C. albicans* SC5313 strain when cannabidiol was used, which was followed by downregulation of genes involved in the biofilm maintenance and development, and in the maturation of factors associated with the EPS synthesis [20].

## 5. Future Perspective

As a future perspective, phytochemicals can not only be used to control biofilm formation, but also to study how cells react when a modest and controllable rise in oxidative stress is experienced. Furthermore, while there are many recent manuscripts on biofilm prevention and removal, we foresee that this review will give also clues on promoting biofilm growth for biotechnological applications, a research topic of growing interest [140]. Nevertheless, a considerable number of issues remain to be clarified or investigated further [141]. In this respect, the incomparability of studies due to different model microorganisms, experimental conditions, and laboratory methods still limit the understanding of the overall mechanism of action. The pro- or antioxidant effects of some molecules in living systems are difficult to determine, as well as the cascade of events triggered by oxidative stress. For instance, the scientific literature reported different sub-MIC concentrations of phytochemicals for each target microorganism. This finding makes challenging to determine the concentration that causes the perturbation of redox homeostasis and, consequently, the antibiofilm activity of the natural compound. Furthermore, the perturbation of redox homeostasis is not exclusive to a specific cellular compartment and it may occur at the same time in different and multiple locations. Finally, the lack of in vivo assays with individual phytochemicals and the phytochemicals in situ persistence must be thoroughly addressed before the more extensive use of plant-based compounds at sub-MIC doses to contrast biofilm.

Many questions remain to be answered: What is the role of oxidative stress in the antibiofilm capacity of phytochemicals? What are the mechanisms through which the perturbation of redox homeostasis can contribute to the antibiofilm performance of phytochemicals? What are the biochemical/molecular targets of ROS? Which biological compartments are affected by oxidative stress? What are the external factors (e.g., UV radiation, heavy metal, growth conditions) that influence the ROS mode of action?

Given the rapid rate at which our knowledge in this area has increased in recent years, it is likely that answers to many of these questions will be forthcoming in the decades. These answers will undoubtedly help to determine whether ROS are merely consequences of cellular response to treatment or, instead, are central regulators of the phytochemicals’ antibiofilm capacity.

## Figures and Tables

**Table 1:** Selected literature on the effects of phytochemical treatment on biofilms at sub-MICs.

Phytocompound	Target Microorganism/s	Sub-Lethal Concentrations Tested	Prevention vs. Control *	Biofilm Growth and Analyses	Oxidative Stress	Effects of the Presence of the Phytocompound
Vanillin (M.W. = 152.2) [33]	A mixed culture including species of *Comamonas*, Enterobacteriaceae, *Pseudomonas*, *Stenotrophomonas*, *Nakamurella*, *Clostridium*, *Azospira*, *Sphingomonas* and *Ferribacterium*	0.32–1.97 mM (0.05–0.3 mg/mL)	Prevention: 52% biofilm reduction with 1.97 mM (0.3 mg/mL) vanillin Control: no efficacy	Microtiter plates: crystal violet assay; fluorescent concanavalin A (Con A) and amine-reactive fluorescein isothiocyanate (FITC) combined with CLSM to visualize the matrix polysaccharides and the proteins, respectively	Not investigated	Not investigated
Vanillin [34]	*Candida albicans* ATCC 90028	0.41–3.29 mM (0.062–0.5 mg/mL)	Prevention: 33% biofilm reduction with 3.29 mM (500 µg/mL) vanillin	Microtiter plates: XTT assay; microscopy	Not investigated	Inhibition of ergosterol biosynthesis
*cis*,*trans*-nepetalactone (M.W. = 166.22), 1,5,9-epideoxyloganic acid (M.W. = 360.4), rosmarinic acid (M.W. = 360.3) [35]	*Pseudomonas aeruginosa* (ATCC 27853)	0.03 mM *cis*,*trans*-nepetalactone (0.005 mg/mL); 0.09 mM *cis*,*trans*-nepetalactone (0.015 mg/mL); 0.056 mM 1,5,9-epideoxyloganic acid (0.02 mg/mL); 0.014 mM rosmarinic acid (0.005 mg/mL)	Prevention: 32% and 41% biofilms reduction with 0.03 mM (0.005 mg/mL) *cis*,*trans*-nepetalactone and with 0.09 mM (0.015 mg/mL) *cis*,*trans*-nepetalactone, respectively Control: no efficacy	Microtiter plates: crystal violet assay	Not investigated	Not investigated
Vitisin B (M.W. = 906.9) [36]	*Escherichia coli* O157:H7 and *P. aeruginosa*	0.0055–0.055 mM (5–50 μg/mL)	Prevention: 90% reduction in *E. coli* biofilm with 0.0055 mM (5 μg/mL) vitisin B Control: not investigated	Microtiter plates: crystal violet assay; GFP cells combined with CLSM	Not investigated	Overexpression of motility genes (*fliA*, *flhD*, *motB* and *qseB*) Reduction in fimbriae
Cyanidin (M.W. = 287.2) [37]	*Klebsiella pneumoniae* strain PUFST23	0.17–0.52 mM (50–150 μg/mL)	Prevention: 72.43% biofilm reduction with 0.52 mM (150 μg/mL) cyanidin Control: not investigated	Microtiter plates: crystal violet assay; acridine orange staining combined with CLSM	Not investigated	Inhibition of quorum sensing activity
Ascorbic acid (M.W. = 176.1) [19]	*P. aeruginosa* clinical isolates	0.11–1.77 mM (19.5–312.5 μg/mL)	Prevention: 100% biofilm reduction with all the concentrations of ascorbic acid Control: not investigated	Microtiter plates: crystal violet assay	Not investigated	Dowregulation of the biofilm-forming genes *lasR* and *pelA*
Ascorbic acid [38]	*K. pneumoniae* strain KP1088 and HvKP3	22.7–181.7 mM (4–32 mg/mL)	Prevention: significant biofilms reduction with 181.7 mM (32 mg/mL) ascorbic acid Control: not investigated	Microtiter plates: crystal violet assay; CLSM	Increased accumulation of ROS	Suppression the biofilm exopolysaccharide and inhibition of the efflux pump
Quercetin (M.W. = 302.2) [39]	*Enterococcus faecalis* MTCC 2729	0.21–0.85 mM (64–256 mg/L)	Prevention: -95% biofilm reduction with 0.85 mM (256 mg/L) quercetin Control: not investigated	Microtiter plates: crystal violet assay; SEM; CLSM	Not investigated	Suppression of proteins related to translation and elongation factors
Berberine (M.W. = 336.4) [40]	*P. aeruginosa* PAO1 and *Salmonella enterica* sv. Typhimurium	0.11–1.86 mM (0.625 and 0.038 mg/mL)	Prevention: 71.7% reduction in *P. aeruginosa* PA01 biofilm with 1.86 mM (0.625 mg/mL) berberine; 31.2% reduction in *S. Typhimurium* biofilm with 0.056 mM (0.019 mg/mL) berberine Control: not investigated.	Microtiter plates: crystal violet assay; acridine orange staining combined with CLSM.	Not investigated	Interaction with the quorum sensing signal receptors, *LasR* and *RhlR*. Inhibition of swimming and swarming motility in *P. aeruginosa* PA01
Berberine [41]	*Staphylococcus epidermidis* ATCC 12228, ATCC 35984 and strain SE243	0.045–0.19 mM (15–75 μg/mL)	Prevention: 100% biofilm reduction with 0.19 mM (75 μg/mL) berberine Control: not investigated	Microtiter plates: crystal violet assay; acridine orange staining combined with CLSM; SEM	Not investigated	Not investigated
*trans*-cinnamaldehyde (M.W. = 132.2); carvacrol (M.W. = 150.2); thymol (M.W. = 150.2); eugenol (M.W. = 164.2) [42]	*Listeria monocytogenes* ATCC 19115, Scott A and Presque-598	0.75 mM *trans*-cinnamaldehyde; 0.65 mM carvacrol; 0.50 mM thymol; 2.5 mM for eugenol	Prevention: All the molecules inhibited biofilm formation Control: All the molecules inactivated fully formed biofilms	Microtiter plates: plate count; SYTO and propidium iodide staining combined with CLSM	Not investigated	Downregulation of genes involved in the attachment (*flaA*, *fliP*, *fliG*, *flgE*, *motA*, *motB*), quorum sensing (*agrA*, *agrB*, *agrC*), stress response (*dnaK*) and transcriptional regulation
Cinnamaldehyde [43]	*Vibrio harveyi* strains, *V. anguillarum* LMG 4411, *V. vulnificus* LMG 16867	0.150 mM	Prevention: 26% and 27% reduction in LMG 4411 and LMG 16867, respectively with 0.15 mM cinnamaldehyde Control: not investigated	Microtiter plates: crystal violet assay; resazurin assay; Calcofluor white staining combined with a fluorometer	Not investigated	Decreasing in DNA-binding ability of *LuxR*, a key factor that drives quorum sensing
*Trans*-cinnamaldehyde [44]	*Cronobacter sakazakii* ATCC 51329, CS 415, CS 4581, CS 4586 and CS 4603	560 and 750 mM	Prevention: 4.0 and 3.0 log CFU/mLwith 750 mM *trans*-cinnamaldehyde Control: not investigated	Microtiter plates: crystal violet assay Tube with different coupons: plate count	Not investigated	Downregulation of *rpoS*, chaperonins, *phoP/Q*, outer membrane porins, and osmolyte transporter genes
Coumarin (M.W. = 146.1); umbelliferone (M.W. = 162.14) [45]	*E. coli* O157:H7 (ATCC43895)	0.34 mM coumarin (50 μg/mL); 0.31 mM umbelliferone (50 μg/mL)	Prevention: 80% and 90% biofilms reduction with 0.34 mM coumarin and 0.31 mM umbelliferone, respectively Control: not investigated	Microtiter plates: crystal violet assay; GFP cells and CLSM	Not investigated	Downregulation genes involved in curli formation (*sgA* and *csgB*) and motility (*flhD* and *motB*)
6-methylcoumarin (M.W. = 160.2) [46]	*P. aeruginosa* PAO1	0.38–1.56 mM (62–250 μg/mL)	Prevention: biofilm inhibition with 0.78 mM (125 μg/mL) 6-methylcoumarin Control: not investigated	Microtiter plates: crystal violet assay; acridine orange staining combined with CLSM	Not investigated	Reduction in motility and quorum sensing activity
Shikonin (M.W. = 290.3) [15]	*Listeria monocytogenes* ATCC 19115 and ATCC 15313	0.011–0.0013 mM (0.39–3.13 μg/mL)	Prevention: 50% biofilm reduction with all the concentrations of shikonin after 5 days of incubation Control: not investigated	Microtiter plates: crystal violet assay; Field-emission scanning electron microscopy (FESEM)	Not investigated	Downregulation quorum sensing, flagellum formation, and autoregulatory alternative sigma factor *SigB*
Zosteric acid (M.W. = 244.2) [23]	*Candida albicans* strain SC5314	0.041 mM (10 μg/mL)	Prevention: 80% biofilm reduction with 0.041 mM (10 μg/mL) zosteric acid Control: 80% biofilm disruption with 0.041 mM (10 μg/mL) zosteric acid	Microtiter plates: Fluorescent Brightener staining coupled with a fluorometer CDC bioreactor: plate count, FUN1 staining and microscopy	Not investigated	Not investigated
Piperine (M.W. = 285.3) [47]	*Staphylococcus aureus* (MTCC 96)	0.028–0.11 mM (8–32 µg/mL)	Prevention: 52% biofilm reduction with 0.028 (8 µg/mL) and 0.056 mM (16 µg/mL) piperine Control: 39% biofilm disruption with 0.056 mM (16 µg/mL) piperine	Microtiter plates: crystal violet assay; protein quantification; acridine orange staining combined with fluorescence microscopy	Increased accumulation of ROS	Decrease in bacterial motility
Cannabidiol (M.W. = 314.5) [20]	*Candida albicans* SC5313	0.0050–0.32 mM (1.56 to 100 µg/mL)	Prevention: 39% biofilm reduction with 0.020 mM (6.25 µg/mL) cannabidiol Control: 44% biofilm disruption with 0.0099 mM (3.12 μg/mL) cannabidiol	Microtiter plates: GFP cells, metabolic activity of the biofilms with MTT assay; Calcofluor White M2R combined with CLSM; mitochondrial function; ATP level	Increased accumulation of ROS	Downregulation of genes involved in biofilm maintenance, development, and maturation of factors associated with EPS synthesis
Myricitrin (M.W. = 318.2); Hesperidin (M.W. = 610.5); Phloridzin (M.W. = 436.4); Myricetin (M.W. = 464.4); Hesperetin (M.W. = 302.3); Phloretin (M.W. = 274.3) [48]	*S. aureus* RN4220 and *S. aureus* SA1199B	Myricitrin 0.0031–0.80 mM; hesperidin 0.0016–0.42 mM; phloridzin 0.0023–0.59 mM; myricetin 0.0022–0.55 mM; hesperetin 0.0033–0.85 mM; phloretin 0.0037–0.93 mM (0.25–256 μg/mL)	Prevention: 50% reduction in RN4220 biofilm with 0.0022 mM myricetin (1 μg/mL) and 0.0037 mM phloretin (1 μg/mL), respectively; 50% reduction in SA1199B biofilm with 0.11 mM (32 μg/mL) hesperetin and 0.069 mM (32 μg/mL) myricetin, respectively Control: not investigated	Microtiter plates: crystal violet assay	Not investigated	Not investigated
Epigallocatechin gallate (M.W. = 458.4) [49]	*Vibrio mimicus*	0.139 mM and 0.279 mM (64 and 128 μg/mL)	Prevention: biofilm reduction with 0.139 mM (64 μg/mL) epigallocatechin gallate Control: not investigated	Microtiter plates: crystal violet assay; FTIC and PI staining combined with CLSM	Increased accumulation of ROS	Inhibition of motility
Epigallocatechin gallate [50]	*E. coli* K12 strains	0.0088–0.87 mM (2.5–400 μg/mL)	Prevention: biofilm reduction with 0.044 mM (12.5 μg/mL) epigallocatechin gallate Control: not investigated	Agar plate: stereomicroscopy; GFP strain and biofilm cryosection; SEM	Not investigated	Effect on the assembly of curli subunits into amyloid fibers, and on the σE cell envelope stress response
Catechin (M.W. = 290.3); Epicatechin (M.W. = 290.3); Gallocatechin (M.W. = 306.3), Epigallocatechin (M.W. = 306.3); Catechin gallate (M.W. = 442.4); Epicatechin gallate (M.W. = 442.4); Gallocatechin gallate (M.W. = 458.4); Epigallocatechin gallate [51]	*Eikenella corrodens* 1073	0.1–0.25 mM	Prevention: significant biofilm reduction with 0.1 mM catechin gallate, epicatechin gallate, gallocatechin gallate and epigallocatechin gallate Control: not investigated	Microtiter plates: XTT assay	Not investigated	Not investigated
Purpurin (M.W. = 285.3) [52]	*C. albicans* strain SC5314	0.0035–0.035 mM (1–10 µg/mL)	Prevention: 64% biofilm reduction with 0.035 mM (10 μg/mL) purpurin Control: not investigated	Microtiter plates: XTT assay; SEM	Not investigated	Downregulation of the expression of the hypha-specific genes
Icariin (M.W. = 676.7); Salidroside (M.W. = 300.3); Resveratrol (M.W. = 228.3) [53]	*Propionibacterium acnes* strains: LMG 16711 (isolated from human facial acne in the UK), LMG 16712 (isolated from human acne) and LMG 16715 (isolated from human blood)	Icariin 0.15–1.18 mM (0.01–0.08%); Salidroside 0.67–83.3 mM (0.02–2.5%); Resveratrol 0.88–14 mM (0.02–0.32%)	Prevention: not investigated Control: 70% biofilm reduction with 1.18 mM (0.08%) icariin; 80% biofilm reduction with 14 mM (0.32%) resveratrol	Microtiter plates: resazurin-based viability assay	Not investigated	Not investigated
Esculetin (M.W. = 178.1); Fisetin (M.W. = 286.2) [18]	*S. aureus* strain 8324 and 8325-4, *S. dysgalactiae* subsp. *dysgalactiae* NCTC 4671 and ATCC 27957	Esculetin 0.18–0.72 mM (32–128 µg/mL) Fisetin 0.014–0.056 mM (4–16 µg/mL)	Prevention: 77% reduction in *S. aureus* 8324 with 128 μg/mL esculetin. 85% reduction in *S. aureus* 8325-4 and 65% reduction in *S. dysgalactiae* NCTC 4671 and ATCC 27957 biofilms with 16 µg/mL fisetin Control: not investigated	Microtiter plates: XTT assay; CLSM	Not investigated	Not investigated
Curcumin (M.W. = 368.4) [54]	*Aeromonas sobria*	Free curcumin (35–280 µg/mL) Curcumin liposomes (52.5–420 µg/mL)	Prevention: 52% and 93.4% biofilms reduction with 280 µg/mL free curcumin and 420 µg/mL curcumin liposomes, respectively Control: not investigated	Microtiter plates: XTT assay; SEM; CLSM	Not investigated	Effects on siderophore production, swimming and swarming motility, extracellular proteases, biofilm formation and N-acylhomoserine lactones production
Eugenol (M.W. = 164.2) [55]	*Pseudomonas aeruginosa* strain PAO1 and two clinical isolates of *P. aeruginosa* (RRLP1 and RRLP2)	0.2–0.6 mM	Prevention: 66% reduction in PAO1 biofilm, 68% reduction in RRLP1 biofilm and 64% reduction in RRLP2 biofilm with 0.4 mM eugenol Control: Eugenol treatment reduced biofilm and the extracellular matrix	Microtiter plates: crystal violet assay	Not investigated	Repression of QS associated genes

* Prevention: effect of the phytocompound on biofilm genesis. Control: effect of the phytocompound on pre-formed biofilms.

## Data Availability

United Nations Sustainable Development Goals (SDGs), in particular SDG 3 ‘Good health and well-being’ (https://sdgs.un.org/goals (accessed on 9 December 2022)).
